# Complex Relationships Among Masculine Norms and Health/Well-Being Outcomes: Correlation Patterns of the Conformity to Masculine Norms Inventory Subscales

**DOI:** 10.1177/1557988317745910

**Published:** 2017-12-08

**Authors:** Zachary T. Gerdes, Ronald F. Levant

**Affiliations:** 1 Department of Psychology, The University of Akron, Akron, OH, USA

**Keywords:** Conformity to Masculine Norms Inventory (CMNI) subscales, masculine norms, men’s health and well-being

## Abstract

The Conformity to Masculine Norms Inventory (CMNI) is a widely used multidimensional scale. Studies using the CMNI most often report only total scale scores, which are predominantly associated with negative outcomes. Various studies since the CMNI’s inception in 2003 using subscales have reported both positive and negative outcomes. The current content analysis examined studies (*N* = 17) correlating the 11 subscales with 63 criterion variables across 7 categories. Most findings were consistent with past research using total scale scores that reported negative outcomes. For example, conformity to masculine norms has been inversely related to help-seeking and positively correlated with concerning health variables, such as substance use. Nonetheless, past reliance on total scores has obscured the complexity of associations with the CMNI in that 30% of the findings in the present study reflected positive outcomes, particularly for health promotion. Subscales differed in their relationships with various outcomes: for one subscale they were predominantly positive, but six others were mostly negative. The situational and contextual implications of conformity to masculine norms and their relationships to positive and negative outcomes are discussed.

In the last 30 years, research in men and masculinities has produced a body of literature that has found many negative outcomes correlated with masculine norms and related constructs (Wong & Wester, 2016). Notably, the endorsement of traditional masculinity ideology, conformity to masculine norms, gender role conflict, and gender role stress have been related to depression, anxiety, low self-esteem, stress, decreased relationship satisfaction, increased systolic blood pressure, aggression and violent behavior, substance abuse, alexithymia, negative attitudes toward help seeking, racial bias, sexism, and a number of other concerning variables (see Gerdes, Alto, Jadaszewski, D’Auria, & Levant, 2017; O’Neil, 2012; Wong, Ho, Wang, & Miller, 2016). Parallel to these trends in the literature, men’s health researchers have advocated for positive conceptions of masculinity (e.g., Kiselica, Benton-Wright, & Englar-Carlson, 2016; Kiselica & Englar-Carlson, 2010) and strengths-based approaches (e.g., Mahalik, Good, Tager, Levant, & Mackowiak, 2012; Wong, 2006) in order to be more effective in clinical work with men and to engage men in reconstructing their definition of manhood to be healthier (Levant & Kopecky, 1995).

At least 519 studies have shown relationships between *total scale scores* of multidimensional measures of masculinity in men and detrimental health and well-being outcomes (for reviews, see Gerdes et al., 2017; O’Neil, 2008; Wong et al., 2016). However, others have identified that specific masculine norms (i.e., *subscales* of multidimensional measures related to masculine norms) display both positive and negative outcomes (e.g., Hammer & Good, 2010; Levant, Wimer, & Williams 2011). For example, in the study introducing the Conformity to Masculine Norms Inventory (CMNI; Mahalik et al., 2003), associations between CMNI total scores and specific subscale scores were inversely associated with seeking psychological help. On the other hand, conformity to masculine norms has been related to potentially beneficial outcomes, such as a negative correlation between certain CMNI subscale scores and substance use (Levant et al., 2011) and positive correlations with strengths such as courage, endurance, and other variables (Hammer & Good 2010). This raises the question of whether studies using total CMNI scale scores obscure more complex relationships between conformity to specific masculine norms and men’s health and well-being, and whether specific norms differ in whether they are related to positive or negative outcomes.

Two conceptual perspectives have been proposed which may account for the variability in outcomes associated with conformity to masculine norms: the variable-centered perspective (also known as the predictor-centered perspective; Wong et al., 2016) and the person-centered perspective (Wong, Owen, & Shea, 2012). The variable- or predictor-centered perspective posits that conformity or nonconformity to particular masculine norms may be adaptive or maladaptive depending on the masculine norm being conformed to or resisted. For example, this perspective might encompass the proposition that self-reliance will consistently be related to difficulty with interpersonal relationships (Mahalik, Talmadge, Locke, & Scott, 2005). The person-centered perspective, in contrast, suggests that the (mal)adaptiveness related to conformity to particular masculine norms can vary based on cultural differences (e.g., gender, race, ethnicity, religious identity, sexual orientation). That is, negative consequences of conforming or not conforming to the masculine norm of emotional control (EC) can vary depending on how congruent conformity to this masculine norm is with other identities. For example, there may be fewer negative consequences for Asian American men of EC if controlling one’s emotions aligns with Asian American values (Wong et al., 2016). This perspective aligns closely with recent scholarship suggesting the health outcomes related to conformity to masculine norms are largely culturally, situationally, and contextually dependent (e.g., Addis, Mansfield, & Syzdek, 2010; O’Neil, 2015; Vogel & Health, 2016; Way et al., 2014; Wester, 2008).

Other studies have examined meta-analytic results associated with conformity to masculine norms and mental health outcomes (Wong et al., 2016) as well as reliability generalization (the meta-analytic procedure for synthesizing reliability evidence) of the CMNI (Kivisalu, King, Phillips, & O’Toole, 2015). Limited attention has been given to studies which have focused explicitly on how the correlates of the subscales of the CMNI may display complex, and potentially inconsistent, positive and negative relationships with various outcome variables. Examining patterns of correlational findings using the CMNI subscales can provide a nuanced fund of information that can help tease out how the person-centered perspective in men and masculinities research may relate to health outcomes.

## The Conformity to Masculine Norms Inventory

The CMNI (Mahalik et al., 2003) is a widely used measure (O’Neil, 2012) that assesses conformity to 11 masculine norms of hegemonic masculine culture in the United States: Winning, Emotional Control, Risk-Taking, Violence, Dominance, Playboy, Self-Reliance, Primacy of Work, Power over Women, Disdain for Homosexuality, and Pursuit of Status. The original 94-item CMNI (Mahalik et al., 2003) uses a 4-point Likert scale (0 = *Strongly Disagree* to 3 = *Strongly Agree*). Factor analyses have supported the 11-factor structure, and psychometric analyses, including evidence for validity and reliability, have been reported (Kivisalu et al., 2015; Mahalik et al., 2003).

Although the CMNI is a multidimensional measure of 11 specific masculine norms, in the only meta-analysis of the CMNI in relationship to men’s health conducted to date, less than half of the included studies reported subscale results (Wong et al., 2016). Studies of other masculinity constructs have reported similar results (e.g., Gerdes et al., 2017). This is notable considering that although the dimensionality of the CMNI has been established using confirmatory factor analysis (CFA), it has not yet been assessed whether a more general construct corresponding to the total scale score also could be represented in the CFA model of the CMNI using either a bifactor or a hierarchical model (Kline, 2016). A 46-item shortened version of the CMNI (Parent & Moradi, 2011) has been subjected to such analyses, and the investigators concluded: “…although we found that the bifactor model fit significantly better than the hierarchical model for this instrument, the fit of the bifactor model was borderline adequate in an absolute sense, suggesting that the CMNI-46 could benefit from further psychometric investigation” (Levant, Hall, Weigold, & McCurdy, 2015, p. 499). Total score use for other measures of masculinity has been empirically supported using CFA (e.g., the Male Role Norms Inventory-Short Form; Levant, Hall, & Rankin, 2013), but the empirical basis for relying on total scale score use with the CMNI has not been established. That is a glaring limitation in the studies that rely solely on total (or mean) CMNI scores. Further investigation is thus needed for how CMNI subscale-specific findings are related to outcomes.

This study is designed to fill this gap in the literature by conducting a content analysis of relationships that have been reported between the 11 subscales of the CMNI and dozens of health and well-being variables. The aim is to elucidate both the impact of CMNI subscales on specific categories of outcomes and the meanings of the CMNI subscales. Particularly, results from subscale-specific associations with the CMNI are analyzed, lending empirically derived insight that may add to our knowledge of the outcome-centered, person-centered, and/or situational and contextual nature of masculinities.

## Procedure

### Assembling the Domain

The PsycInfo, PsycNET, and PsycArticles databases were used to assemble the domain of published studies using the CMNI. The search terms “Conformity to Masculine Norms Inventory*” and “CMNI*” were used. Inclusion criteria for studies were studies must have: (a) used the original (94-item) CMNI; shorter versions (e.g., CMNI-46; Parent & Moradi, 2011) were excluded due to the lack of comparability of the subscale structures and recent findings suggesting psychometric limitations, particularly with the CMNI-46 (see Levant et al., 2015); (b) examined correlations with variables other than masculinity measures, as this has been studied elsewhere (Gerdes et al., 2017; O’Neil, 2012); (c) used at least 4 of the 11 subscales of the CMNI in order to maintain some consistency in reporting across studies. Seventeen published studies were identified which met the selection criteria, which included correlates with 63 variables. *N*-sizes ranged from 20 to 1,600. Most studies used samples of university students and/or community members. All studies used either correlation or regression analyses; correlational results were used here. Characteristics of each study are summarized in Table 1.

**Table 1.** Studies Using the Subscales of the Conformity to Masculine Norms Inventory.

Author(s) (publication year)	Sample	*N-*Size	Variable(s) used
Amato (2012)	New England prisoners and detainees	1,600 (men)	Prison inmate violence
Backus and Mahalik (2011)	Self-identified heterosexual women	183 (women)	Feminist identity—revelation Feminist identity—synthesis Feminist identity—active commitment Feminist identity—passive acceptance Feminist identity—embeddedness
Burns, Hough, Boyd, and Hill (2010)	Men with spinal cord injury	116	Erectile functioning Age Depression Social support
Burn and Ward (2005)	College men and women	170 (men)	Relationship satisfaction
Hammer and Good (2010)	Community men	250	Courage
			Grit
			Personal control
			Autonomy
			Endurance
			Resilience
			Self-esteem
			Life satisfaction
Kahn, Brett, and Holmes (2011)	College men	164	Internal motivation to know Intrinsic motivation to accomplish Intrinsic motivation to experience stimulation External motivation introjected Extrinsic motivation external External motivation identified
Keiller (2010)	College men identified as “completely heterosexual”	104	Attitudes toward gay men Religious fundamentalism Attitudes toward lesbian women
Levant et al. (2011)	College men (2011)	323	Avoidance of anger/stress
			Avoidance of substance use
			Proper use of health care resources
Limiñana-Gras, Sánchez-López, Saavedra-San, and Corbalán-Berná (2013)	Male nurses	98	Alcohol consumption
			Medical ailments
			Self-perceived poor health
			Medicine consumption
			Doctor visits
			Work satisfaction
Liu and Iwamoto (2007)	Asian American men from the community	154	Alcohol use
			Binge drinking
			Marijuana use
			Cocaine use
			Other substance use
			Peer substance use
Locke and Mahalik (2005)	College men	254	Sexually aggressive behavior Rape myth acceptance Athletic involvement Alcohol use
Mahalik, Levi-Minzi, and Walker (2007)	Australian men	253	Health promoting behavior
Mahalik and Rochlen (2006)	College men	153	Talk to partner (response to depression)
			Talk to mental health professional (response to depression)
			Exercise or workout (response to depression)
			Have a few drinks (response to depression)
Sánchez-López, Cuellar-Flores, and Dresch (2012)	College men from Madrid, Spain	226	Alcohol consumption
Schopp, Good, Mazurek, Barker, and Stucky (2007)	Male spinal cord injury patients	20	Satisfaction with life
			Functional independence
Tager and Good (2005)	Italian male college students in Italy	152	Self-acceptance
			Positive relations with others
Ward and Cook (2011)	College men	154	Religious commitment
			Religious fundamentalism
			Intrinsic religious orientation
			Extrinsic religious orientation

### Content Analysis

After variables were identified, content analysis (Krippendorff, 2003) was used to code correlates and group them into the following categories: Substance Use, Health Promotion, Religiousness, Motivation, Attitudes and Beliefs about Gender, Sex, and Sexual Orientation, Character Strengths and Satisfaction, and Interpersonal Variables. Finally, drawing on categorizations used in reviews of other measures of masculinity-related constructs (e.g., O’Neil, 2008), results were labeled as showing “positive,” “negative,” or “other” outcomes related to men’s health and well-being.

## Results

As reported in Table 2, there were 219 significant findings, with 12 to 31 findings per subscale and 13 to 47 findings per criterion category. There were mixed correlational patterns for most categories and subscales. Attitudes and beliefs about gender, sex, and sexual orientation, substance use, and interpersonal variables had the greatest percentages of negative outcomes (76.5–100%). In contrast, health promotion and religiousness had greater percentages of positive outcomes (53.8–61.8%). A large minority of findings (66, 30%) identified CMNI subscales positively associated with character strengths and satisfaction, progressive views of gender, motivation, religiousness, health promotion, and negatively associated with problematic substance use. A discussion of results with each subscale follows.

**Table 2.** Male Participants’ Correlates of Conformity to Masculine Norms With Criterion Variables.

CMNI subscale findings	Winning	Emotional Control	Risk-Taking	Violence	Power Over Women	Dominance	Playboy	Self-Reliance	Primacy of Work	Disdain for Homosexuals	Pursuit of Status	Total findings
***Substance use***												
Alcohol use^10^	.20*	−.20*	.17*	.17*			.20*				.29**	
Binge drinking^10^	.19*	−.18*					.29**			.23**		
Alcohol use^11^	.28***	.16*	.26***	.29***	.33***	.26***	.41***	.27***		.23***	.25***	
Alcohol consumption^14^						.13*	.20**					
Alcohol consumption (nurses)^9^				.21*			.32**				.23*	
Cigarette use^14^										−.15*		
Marijuana use^10^							.18*				.17*	
Cocaine use^10^							.18*					
Other substance use^10^							.19*					
Peer substance use^10^	−.19*		.37**				.21**	−.24**			.23**	
Avoidance of substance use^8^	.21**						−.25***					
Have a few drinks (response to depression)^13^				.21**	.30***	.22**	.32***					
**Positive associations with substance use**	**3**	**1**	**3**	**4**	**2**	**3**	**11**	**1**		**2**	**5**	**35**
**Negative associations with substance use**	**2**	**2**						**1**		**1**		**6**
***Health Promotion***	***Winning***	***Emotional Control***	***Risk-Taking***	***Violence***	***Power Over Women***	***Dominance***	***Playboy***	***Self-Reliance***	***Primacy of Work***	***Disdain for Homosexuals***	***Pursuit of Status***	***Total findings***
Medical ailments (nurses)^9^										−.27**		
Self-perceived poor health (nurses)^9^		−.30**										
Functional independence (spinal cord injury patients)^15^		.57*				.54*						
Avoidance of anger/stress^8^		.18*						−.22***	.14**			
Preventative self-care^8^									.12*			
Proper use of health-care resources^8^			−.13*				−.17**					
Athletic involvement^11^	.27***											
Erectile functioning^3^									.23*			
Medicine consumption (nurses)^9^				−.20*								
Doctor visits (nurses)^9^		−.20*	.24*			−.20*						
Age^3^							−.28**	−.28**				
Depression^3^		.27**						.43**				
Talk to mental health professional (response to depression)^13^		−.22**		−.20*				−.19*				
Exercise or workout (response to depression)^13^	.34***			.27**					.19*	.21**		
Health promoting behavior^12^	.16**	.15*	.22***	.26***	.21***	.16*	.29***	.24***				
**Positive associations with health promotion**	**3**	**3**	**2**	**2**	**1**	**2**	**1**	**1**	**4**	**2**		**21**
**Negative associations with health promotion**		**4**	**1**	**1**	**0**	**1**	**2**	**4**				**13**
***Religiousness***	***Winning***	***Emotional Control***	***Risk-Taking***	***Violence***	***Power Over Women***	***Dominance***	***Playboy***	***Self-Reliance***	***Primacy of Work***	***Disdain for Homosexuals***	***Pursuit of Status***	***Total findings***
Religious commitment^17^		−.31***		−.24**			−.36***			.26***		
Religious fundamentalism^17^					.28***		−.24*			.46***		
Religious fundamentalism^7^										.28**		
Intrinsic religious orientation^17^		−.32***					−.29***			.36***		
Extrinsic religious orientation^17^	.22*									.26***		
**Positive associations with religiousness**	**1**				**1**					**5**		**7**
**Negative associations with religiousness**		**2**		**1**			**3**					**6**
***Motivation***	***Winning***	***Emotional Control***	***Risk-Taking***	***Violence***	***Power Over Women***	***Dominance***	***Playboy***	***Self-Reliance***	***Primacy of Work***	***Disdain for Homosexuals***	***Pursuit of Status***	***Total findings***
Internal motivation to know^6^		−.22**		−.23**	−.15*			−.31**	.31**			
Intrinsic motivation to accomplish^6^			−.15*	−.19*	−.18*		−.20*	−.26**	.37**			
Intrinsic motivation to experience stimulation^6^	−.17*	−.23**		−.24**				−.19*	.34**			
External motivation introjected^6^		−.20**				.21**		−.27**	.20**			
Extrinsic Motivation external^6^						.32**						
External motivation identified^6^				−.20*		.21**	−.15*	−.16*	.19*			
**Positive associations with motivation**						**3**			**5**			**8**
**Negative associations with motivation**	**1**	**3**	**1**	**4**	**2**		**2**	**5**				**18**
***Attitudes and beliefs about gender, sex and sexual orientation***	***Winning***	***Emotional Control***	***Risk-Taking***	***Violence***	***Power Over Women***	***Dominance***	***Playboy***	***Self-Reliance***	***Primacy of Work***	***Disdain for Homosexuals***	***Pursuit of Status***	***Total findings***
Feminist identity-revelation^2^		−.29***		−.20**	−.24***					−.19*		
Feminist identity-synthesis^2^		−.23**		−.22**	−.37***		−.27***	−.27***	−.19*		.19**	
Feminist identity-active commitment^2^		−.32***		−.28***	−.49***	−.25***	−.19**	−.36***	−.15*	−.19*		
Feminist identity-passive acceptance^2^	.24***	.23**	−.18*		.46***	.28***		.23**		.36***		
Feminist identity-embeddedness^2^		−.19*		−.16*	−.23**			−.21**		−.19*		
Attitudes toward gay men^7^					.34***					.44***		
Attitudes toward lesbian women^7^			−.29*	−.21*			−.29**	−.21*		.34***		
Rape myth acceptance^11^	.16*	.16*		.20**	.41***	.28***	.26***	.14*	.13*	.33***		
**Associations with traditional and regressive views of gender, sex, and sexual orientation**	**2**	**5**		**4**	**6**	**3**	**3**	**4**	**3**	**6**		**36**
**Associations with progressive views of gender, sex, and sexual orientation**		**1**	**2**	**2**	**1**		**1**	**2**		**1**	**1**	**11**
***Character strengths and satisfaction***	***Winning***	***Emotional Control***	***Risk-Taking***	***Violence***	***Power Over Women***	***Dominance***	***Playboy***	***Self-Reliance***	***Primacy of Work***	***Disdain for Homosexuals***	***Pursuit of Status***	***Total findings***
Courage^5^		−.16*	.32***	.14*		.15*		−.15*			.17**	
Grit^5^								−.14*			−.18**	
Personal control^5^	−.14*	−.21**						−.28***				
Autonomy^5^	−.17**	−.15*						−.25***				
Endurance^5^	.17**		.22***									
Resilience^5^		−.26***	.19**					−.26***			.18**	
Self-esteem^5^		−.27***						−.30**			.15*	
Life satisfaction^5^		.19**						−.17**				
Satisfaction with life (spinal cord injury patients)^15^				.52*								
Self-acceptance^16^	.20*			−.17*							.18*	
Work satisfaction (nurses)^9^						−.24*					−.23*	
**Positive associations with strengths/satisfaction**	**2**	**1**	**3**	**2**		**1**					**4**	**13**
**Negative associations with strengths/satisfaction**	**2**	**5**		**1**		**1**		**7**			**2**	**18**
***Interpersonal variables***	***Winning***	***Emotional Control***	***Risk-Taking***	***Violence***	***Power Over Women***	***Dominance***	***Playboy***	***Self-Reliance***	***Primacy of Work***	***Disdain for Homosexuals***	***Pursuit of Status***	***Total findings***
Positive relations with others^16^		−.46**						−.32**				
Relationship satisfaction^4^							−.40***					
Prison inmate violence^1^	.36**	.25**	.43**	.55**	.29**	.36**	.36**	.40**		.29**	.21**	
Sexually aggressive behavior^11^	.20**		.27***	.17**	.33***	.24***	.37***	.15*		.21**		
Social support^3^		−.32**						−.39**				
Talk to partner (response to depression)^13^		−.25**			−.27**	−.18*	−.19*					
**Associations with positive interpersonal relationships/behavior**												**0**
**Associations with negative interpersonal relationships/behavior**	**2**	**4**	**2**	**2**	**3**	**3**	**4**	**4**		**2**	**1**	**27**
***Summary***	***Winning***	***Emotional Control***	***Risk-Taking***	***Violence***	***Power Over Women***	***Dominance***	***Playboy***	***Self-Reliance***	***Primacy of Work***	***Disdain for Homosexuals***	***Pursuit of Status***	***Total findings***
Total findings per subscale	18	31	14	23	16	17	27	29	12	19	13	219
Findings of negative outcomes	9	24	7	17	13	11	25	25	3	10	8	153
Findings of positive outcomes	8	7	7	6	3	6	2	4	9	9	5	66
Other (motivation variables)	1	3	1	4	2	0	2	5	0	0	0	18

*Note.* ^1^Amato (2012). ^2^Backus and Mahalik (2011). ^3^Burns et al. (2010). ^4^Burn and Ward (2005). ^5^Hammer and Good (2010). ^6^Kahn et al. (2011). ^7^Keiller (2010). ^8^Levant et al. (2011). ^9^Limiñana-Gras et al. (2013). ^10^Liu and Iwamoto (2007). ^11^Locke and Mahalik (2005). ^12^Mahalik et al. (2007). ^13^Mahalik and Rochlen (2006). ^14^Sánchez-López et al. (2012). ^15^Schopp et al. (2007). ^16^Tager and Good (2005). ^17^Ward and Cook (2011).

* *p* < .05. **p < .01. ***p < .001.

### Winning

The relationship between Winning and substance use is somewhat unclear. It was positively associated not only with increased alcohol use and binge drinking but also with avoidance of substance use and negatively with peer substance use. These conflicting findings may reflect design and sample differences across multiple studies. Winning was positively associated with endurance and self-acceptance, as well as athletic involvement and exercise. Results indicate that a competitive mindset may foster exercise and endurance. On the other hand winning was negatively associated with personal control, autonomy, and positively associated with rape myth acceptance and sexually aggressive behavior.

### Emotional Control

EC had mixed findings in health promotion and substance use. EC was negatively related to alcohol use and binge drinking. It was also related to the avoidance of anger/stress and to depression, suggesting that controlling one’s emotions can contribute to the control of anger and stress, but that this might lead to depression. This is notable since EC was negatively related to talking to a mental health professional in response to depression. In addition, considering that conforming to the norm of EC may make men “emotionally distant” (Mahalik et al., 2005, p. 662), it is not surprising that it would be related to fewer positive relations with others and decreased communication with one’s partner. EC was inversely related to many positive variables, such as courage, autonomy, resilience, self-esteem, and personal control, yet curiously, it also related positively to life satisfaction. The inverse relationship between emotional control and personal control is puzzling.

### Risk Taking

Findings associated with Risk-Taking were largely mixed. For example, it was related not only to alcohol use and not using health care resources properly but also to health promotion. Risk-Taking was positively associated with three strength variables: courage, endurance, and resilience. On the other hand, it was associated with sexually aggressive behavior.

### Violence

Violence was associated with alcohol use, courage, and exercising in response to depression, suggesting violence requires courage and may be abetted by alcohol use. Violence had inverse relationships with variables that imply an involvement with more vulnerable emotional processes: religious commitment, self-acceptance, and talking to a mental health professional in response to depression. This suggests that conforming to the violence norm may be a way of denying vulnerability. Violence was not only related to less motivation, but also to health-promoting behavior.

### Power Over Women and Disdain for Homosexuals

Power Over Women and Disdain for Homosexuals were most prominently associated with regressive views of gender, sex, and sexual orientation. These two subscales also had the largest correlations with rape myth acceptance across subscales (*r* = .41 and .33, *p* < .001, respectively). Both were related to sexually aggressive behavior. Disdain for Homosexuals was the only subscale to be positively correlated with all four aspects of religiousness, including Religious Fundamentalism, which is consistent with research finding a relationship between religious fundamentalism and sexual prejudice (McCleary, Quillivan, Foster, & Williams, 2011; Mellinger & Levant, 2014; Rowatt et al., 2013).

### Dominance

Dominance was associated with courage, rape myth acceptance, sexually aggressive behavior, external motivation, and prison inmate violence. This subscale also had an inverse relationship to work satisfaction for male nurses and talking with one’s partner in response to depression. Dominance was positively related to three indices of alcohol use. Besides courage, Dominance was related to few positive outcomes.

### Playboy

Playboy was associated with 11 indices of substance use, increased rape myth acceptance, sexually aggressive behavior, prison inmate violence, and decreased relationship satisfaction and talking with one’s partner. The Playboy subscale was predominantly associated with negative outcomes.

### Self-Reliance

Self-reliance was related to a total of 29 variables—16 of which were negative associations with character strengths and satisfaction (7), motivation (5), and negative aspects of interpersonal relationships (4). Five findings linked Self-Reliance positively with substance use and negatively with health promotion, but two findings (decreased peer substance use and increased health promoting behavior) countered this. In short, correlates of Self-Reliance seem largely negative with few exceptions.

### Primacy of Work and Pursuit of Status

Primacy of Work and Pursuit of Status were associated with the fewest significant findings across all subscales. Primacy of Work had only positive outcomes; it was positively correlated with five motivation variables and with four indices of health promotion. Pursuit of status was associated with five indices of substance use, and negatively associated with grit and work satisfaction. However, it was also related to courage, resilience, self-esteem, self-acceptance, and feminist identity–synthesis.

## Discussion

Results from the present study indicate that subscale findings should always be reported in studies examining conformity to masculine norms, as they reveal complex relationships that may be masked when only total scores are reported, which (as discussed above) does not have empirical support. Nonetheless, prior research using total scores which report predominantly negative outcomes is supported by this content analysis of subscale scores, in that most of the present findings reflected negative outcomes (153, 69.9%). This is clearer for some health and well-being outcome criteria than for others: regressive views of gender, sex, and sexual orientation (76.6% of the findings reflected negative outcomes), substance use (82.9%), and less positive interpersonal relationships (100%). Nonetheless, reliance on total scores has obscured part of the picture, in that 66 findings (30%) in the present study reflected positive outcomes. For example, the relationship between the CMNI subscales and health promotion is largely positive.

Subscales also differed in terms of their outcomes. While one subscale (Primacy of Work) was predominantly associated with positive outcomes, four others had a fairly equal balance of positive and negative outcomes (Winning, Risk-Taking, Pursuit of Status, and Disdain for Homosexuals). However, six subscales were mostly associated with negative outcomes (Emotional Control, Violence, Power over Women, Dominance, Playboy, and Self-Reliance).

While relationships between some subscales and outcome areas are fairly clear, others defy immediate explanation. In these cases, the contradictory results could be further investigated by drawing on the contingent and contextual nature of masculine norms (Addis et al., 2010).

The outcome for any man who conforms to particular masculine norms may be situationally dependent (Isacco, 2015). Hence, one way to explicate seemingly contradictory findings (such as Winning being positively associated with increased alcohol use but also negatively associated with avoidance of substance use) would be to manipulate the directions for completing the scale by referencing specific situations. This would require identifying the contexts in which conforming to certain masculine norms may be beneficial or detrimental. The present findings may provide a point of departure for research examining masculinity-in-context, in which contextual variables may moderate the relationships between conformity to masculine norms and various outcomes. In the meantime, conformity to masculine norms must not be regarded as wholly negative.

These results also inform considerations of the harmful associations with masculine norms as either variable-centered (i.e., particular masculine norms being associated consistently with either positive or negative outcomes) or person-centered (i.e., positive or negative outcomes related to masculine norms vary depending on individual differences, contextual influences, and cultural factors). If outcomes associated with subscale scores display relationships that differ from total score findings, the differential impacts that particular masculine norms are having on men’s health may be overlooked when only total scores are considered. Researchers should be encouraged to report and compare subscale findings related to conformity to masculine norms.

More specifically, future research must examine two things: (a) how individual differences, contextual influences, and cultural factors moderate and/or mediate associations between masculine norms and outcomes (Wong et al., 2016) and (b) the ways in which men actually perceive their conformity to particular norms as “masculine” (Isacco, 2015). Regarding the former, more experimental studies as well as moderation and mediation analyses in correlational studies are warranted. Qualitative research is also needed which examines how and why men perceive particular beliefs or behaviors as personally masculine to them. In addition, more meta-analyses in the psychological study of men and masculinities are required to further illuminate patterns of health outcomes that are related to total and subscale scores of various masculinity measures. Meta-analyses for the most commonly used constructs using of masculinity-related measures may be good places to start—namely with gender role conflict using the Gender Role Conflict Scale (O’Neil, Helms, Gable, David, & Wrightsman, 1986), conformity to masculine norms (examined here) with the CMNI (Mahalik et al., 2003), and “traditional” masculinity ideology with the Male Role Norms Inventory (Levant et al., 1992). While one meta-analysis has been completed on the CMNI to date (Wong et al., 2016), it was limited to mental health-related outcomes. Future meta-analyses should be more comprehensive in scope. Results from the current study suggest future studies and meta-analyses using the CMNI should examine and compare total and subscale scores alike.

The crux of improving men’s mental and physical health relies on men’s ability to do two things simultaneously: increase beliefs and behaviors that promote health (Wong et al., 2016) while performing their gender identity in positive ways (Burkley, Wong, & Bell, 2016). In other words, men must be able to “feel like a man” in ways congruent with beliefs and behaviors that promote health. If studies on men and masculinities continue to rely on total scores of measures of masculine norms, solutions for creating pathways that can catalyze this congruence will be stymied.

### Limitations

The following limitations of the present content analysis must be noted. First, as this study was meant to only summarize previous findings, the methodological adequacy of the included studies was not critically assessed. Thus, findings of studies are compared in which participants and procedures may differ across studies. For example, results from studies using community participants were compared to results using college students. Further, demographic diversity of participants was not analyzed outside of identifying the type of sample (e.g., college or community). In addition, only correlational data was used in this study for the sake of clarity and brevity. However, findings in the original studies included additional results using multiple linear regression. Of course, correlational relationships cannot be assumed to be causal. Lastly, because the current study examined results specific to the subscales on the original 94-item version of the CMNI for psychometric reasons, findings cannot be generalized to other forms of the CMNI. In addition, this analysis was intentionally limited to published studies which used the 94-item CMNI and 4 or more CMNI subscales, but future investigation might examine studies which used other versions of the CMNI, fewer subscales, and are unpublished, including dissertations and theses.

## Conclusion

Over 500 studies conducted over at least three decades have examined outcomes related to conformity to and belief in specific identified masculine norms and related constructs in men (Wong & Wester, 2016). Hundreds of findings have evidenced harmful health outcomes associated with masculine norms (Gerdes et al., 2017; Wong et al., 2016). However, knowing how and in what ways these relationships serve to promote or risk health will depend on future research that compares total scores to subscale scores of measures of masculine norms while diversifying research methods and statistical analyses (Wong & Horn, 2016). Experimental methods, mediation and moderation regression studies, meta-analyses, and qualitative research will further illuminate the complex relationship between masculine norms and men’s health.

With this growing foundation, preventive and therapeutic interventions can be designed for men that will aid them to authentically perform their personal masculinities while maintaining their health. Men can feel like men while being mentally and physically healthier. Norms, situations, and contexts influencing the relationship between certain masculine norms and harmful or health-promoting factors must be further explored.
